# Chronic Internal Exposure to Low Dose ^137^Cs Induces Positive Impact on the Stability of Atherosclerotic Plaques by Reducing Inflammation in ApoE^-/-^ Mice

**DOI:** 10.1371/journal.pone.0128539

**Published:** 2015-06-05

**Authors:** Clélia Le Gallic, Yohann Phalente, Line Manens, Isabelle Dublineau, Marc Benderitter, Yann Gueguen, Stephanie Lehoux, Teni G. Ebrahimian

**Affiliations:** 1 IRSN, Institut de Radioprotection et de Sûreté Nucléaire, Laboratoire de RadioToxicologie Experimentale, 92262, Fontenay-aux-Roses, France; 2 Lady Davis Institute, McGill University, Montreal, Canada; University of Amsterdam Academic Medical Center, NETHERLANDS

## Abstract

After Chernobyl and Fukushima Daï Chi, two major nuclear accidents, large amounts of radionuclides were released in the environment, mostly caesium 137 (^137^Cs). Populations living in contaminated territories are chronically exposed to radionuclides by ingestion of contaminated food. However, questions still remain regarding the effects of low dose ionizing radiation exposure on the development and progression of cardiovascular diseases. We therefore investigated the effects of a chronic internal exposure to ^137^Cs on atherosclerosis in predisposed ApoE^-/-^ mice. Mice were exposed daily to 0, 4, 20 or 100 kBq/l ^137^Cs in drinking water, corresponding to range of concentrations found in contaminated territories, for 6 or 9 months. We evaluated plaque size and phenotype, inflammatory profile, and oxidative stress status in different experimental groups. Results did not show any differences in atherosclerosis progression between mice exposed to ^137^Cs and unexposed controls. However, ^137^Cs exposed mice developed more stable plaques with decreased macrophage content, associated with reduced aortic expression of pro-inflammatory factors (CRP, TNFα, MCP-1, IFNγ) and adhesion molecules (ICAM-1, VCAM-1 and E-selectin). Lesions of mice exposed to ^137^Cs were also characterized by enhanced collagen and smooth muscle cell content, concurrent with reduced matrix metalloproteinase MMP8 and MMP13 expression. These results suggest that low dose chronic exposure of ^137^Cs in ApoE^-/-^ mice enhances atherosclerotic lesion stability by inhibiting pro-inflammatory cytokine and MMP production, resulting in collagen-rich plaques with greater smooth muscle cell and less macrophage content.

## Introduction

After the accidents at Chernobyl (1986) and Fukushima (2011), large amounts of caesium 137 (^137^Cs) were released into the environment **[1]**. ^137^Cs is the main fission product of uranium and plutonium within nuclear reactors. It is a radionuclide which bears a low chemotoxicity but high radiotoxicity, mainly related to emission of β and γ rays **[2]**. Because ^137^Cs has a 30 year half-life, it remains one of the major sources of exposure for the population in contaminated territories **[3]**. Several studies have reported that the youngest children in the contaminated areas exposed to the Chernobyl accident are developing thyroid cancer, bone disorders **[4]**, as well as nervous and digestive system disorders **[5]**. The cardiovascular system may also be targeted.

The two major contributors to cardiovascular diseases (CVD), myocardial infarction and stroke, arise as a result atherosclerotic plaque rupture. Atherosclerosis is a chronic inflammatory pathology of large and medium arteries **[6]**. In the course of the atherogenic process, oxidized low-density lipoproteins enter the vascular wall and induce an inflammatory reaction. As a result, endothelial cells express adhesion molecules such as E-selectin (ESel), intracellular adhesion molecule-1 (ICAM-1), vascular cell adhesion molecule-1 (VCAM-1) that are necessary for the adhesion and diapedesis of monocytes. Concurrently, a number of pro-inflammatory cytokines such as tumor necrosis factor alpha (TNFα), interferon gamma (IFNγ), and monocyte chemo attractant protein-1 (MCP-1) are released, promoting monocyte chemotaxis. All of these factors contribute to plaque progression **[7]**. Monocytes within the lesion differentiate into macrophages which incorporate the oxidized low-density lipoproteins and become foam cells. These lipid-laden cells have low structural strength and contribute to plaque fragility. On the contrary, vascular smooth muscle cells (VSMC) are the main components of atherosclerotic plaques responsible for promoting plaque stability; they produce collagen and other extracellular matrix proteins that constitute the fibrous cap **[8]**, which protects lesions from rupture **[9]**. It is well documented that vulnerable plaques have high expression of some metalloproteinases (MMP 2, 3, 8 and 13) that degrade matrix components and are responsible for plaque destabilization and rupture **[10]**. The production of these MMPs by VSMCs and macrophages is regulated by inflammation **[6]**. Thus, most clinical manifestations of atherosclerosis, which are due to plaque rupture, depend on the change in balance between VSMCs and collagen buildup on the one hand, and foam cell accumulation and fibrous cap degradation by MMPs on the other hand. Inflammation is the primary underlying cause of atherosclerotic plaque instability and rupture **[6]**, and C-reactive protein (CRP), a marker of inflammation, has become the prototypic risk marker for CVD **[11]**.

Exposure to high doses of ionizing radiations increases the risk of CVD due to damages to the heart structure and vessels **[12–14]**. Liquidators who took part in the repair at Chernobyl are reported to have loss of vascular tone, amplified venous return, and increased myocardial contractility **[15]**. High levels of ^137^Cs were observed in the hearts of adults and children living in the contaminated areas of Belarus, and they may be related to a wide variety of changes to this organ, including cardiovascular symptoms, electrocardiography alterations, and hypertension **[16]**, although the elevated blood pressure may be related to not only to the ^137^Cs but also perhaps to the psychological stress provoked by the accident **[17]**. Studies of atomic bomb survivors in Japan who received a single dose to the whole body of 0 to 4 Gy, showed a dose-related increase in serum lipids, associated with greater CVD risk **[18]**. Some studies have even established a link between radiation exposure and atherosclerosis [19]. In patients with Hodgkin’s disease, radiation therapy was associated with a greater intima-media thickness, an index of atherosclerotic lesion size [20], and with early onset myocardial infarction [21–24]. Similarly, carotid artery atherosclerosis is described as a relevant complication of neck radiotherapy [25–27]. However, irradiating existing atherosclerotic lesions in mice led to smaller, albeit more inflamed plaques [28].

However, it is unclear from previous studies whether an association exists between atherosclerosis and low dose radiation exposure **[29, 30]**, and whether the mechanisms implicated differ from high-dose effects **[31].** These questions are particularly pertinent in the setting where the re-population of contaminated territories is envisioned after a nuclear accident. An experimental study in rats showed that chronic exposure to low doses of ^137^Cs decreased mean arterial blood pressure but increased the expression of angiotensin and brain natriuretic peptide [32], which could have opposite effects on atherosclerosis. However, in diabetic mice, repeated low-dose radiation reduced lipid levels and attenuated inflammation [33], and reduced cardiac expression of multiple inflammatory agents [34]. Likewise, multiple low-dose irradiation of mice reduced inflammatory signalling in a mouse model of asthma [35].

Hence, anti-inflammatory effects of low dose radioactivity were reported in models of chronic inflammatory diseases. We therefore hypothesized that chronic low dose exposure to ^137^Cs would regulate atherosclerosis progression in mice. Genetically predisposed Apolipoprotein E^-/-^ (ApoE^-/-^) mice **[36, 37]** were chronically exposed to 20, 100 or 500 Bq/animal of ^137^Cs administrated through drinking water. Our dosing range (4–100 kBq/l) corresponded to a range spanning 5 times less and 5 times more than the concentration of 20 kBq/l used in previous studies **[38]**. More importantly, our ingestion rates are close to the estimated ingestion rates of 20 to 2100 Bq/day that were calculated to be ingested by humans living in contaminated areas **[39]**. As a whole, ^137^Cs contamination of human tissues was previously found to be in the range of 100 and 2000 Bq/kg, measured in the whole body **[40],** in urine samples **[41]** or in organs at autopsy **[42, 43].** We investigated atherosclerosis progression along with oxidative stress balance, inflammatory status and indices of plaque stability at different stages of the pathology.

## Material and Methods

### Animals

7–8 week-old ApoE^-/-^ male mice were obtained from Charles River Laboratory. Each group was comprised of 10 animals. ApoE^-/-^ mice are homozygous null for a functional ApoE gene on a C57BL/6J background. Apolipoprotein E acts as the main ligand mediating removal of cholesterol enriched chylomicron and very low density lipoprotein remnants from the blood stream and plays an important role in lipoprotein metabolism. These mice develop atherosclerosis when fed with a normal low fat diet. The morphological features of early-stage lesions in ApoE^-/-^ mice are very similar to those found in humans **[44]**. Animals were maintained in a specific-pathogen-free environment and monitored daily. All experiments and procedures were carried out in accordance with the Guide for the Care and Use of Laboratory Animals as published by the French regulations for animal experiments (Ministry of Agriculture Order No. B92-032-01, 2006) with European Directives (86/609/CEE), and approved by the local ethical committee of the Institute for Radiological Protection and Nuclear Safety (Permit Number: P10-11 and thematic number: T29).

### Cs 137 exposure

Animals were separated into 4 groups: ApoE^-/-^ mice receiving either tap water *ad libitum* or tap water *ad libitum* supplemented with 4, 20 or 100 kBq/l of ^137^Cs (^137^CsCl final concentration 5x10^-9^M, CERCA-LEA, Pierrelatte, France), during 6 or 9 months.

### Blood sampling and analysis

Mice were terminally anesthetized by intraperitoneal injection of ketamin/xylazin (Ketamine 500 Virbac, Rompun 2% Bayer). Blood was collected by intracardiac puncture with a heparinised syringe. Blood was centrifuged for 8 minutes at 800 *g* and plasma was harvested and frozen for subsequent analysis. Plasma cholesterol, low and high density lipoproteins (LDL and HDL) levels were determined with an automated spectrophotometric system (Konelab 20, Biological Chemistry Reagents, Thermo Electro Corporation).

### Tissue collection

Hearts (including the aortic root) were separated from the aorta, embedded in optimum cutting temperature medium (OCT, Sakura Fineteck), and snap-frozen on a metal plate that was cooled with liquid nitrogen. Aortas were excised from the aortic arch to the femoral bifurcation and directly snap frozen on liquid nitrogen. The thoracic and abdominal aortas were separated for gene and protein expression analysis.

### Cs 137 measurement

Gamma spectrometry with a gamma counter (Packard Cobra Model II D5003) was used to measure ^137^Cs. The gastrocnemus muscle was counted for 60 minutes, and the count was related to its weight. The detection limit ranged from 4.2 to 12 counts per minute per sample, depending on the mass of the organ tested and the duration of the counting period. The radiation dose received by the muscle was calculated according to models developed for rodents **[45]**, as previously described **[46]**.

### Gene expression

Total RNA from thoracic aortas was extracted. Briefly, aortas, are grinded with a Precellys 24 (Bertin Technologies) using TRI Reagent solution (Sigma-Aldrich). After extraction, we performed RNA purification (RNeasy Mini Kit, Qiagen). RNA quality was checked by measuring the ratio of optical densities at 260 and 280 nm. Real-time qPCR (RT-qPCR) was used to analyse the mRNA levels of inflammatory cytokines and adhesion molecules: CRP, TNFα, MCP-1, IFNγ, ICAM-1, VCAM-1, and ESel. Oxidative stress balance was evaluated by mRNA expression of pro and anti-oxidant enzymes catalase (CAT), heme oxygenase 1 (HO-1), nuclear factor-like 2 (Nrf2), and gluthathione peroxydase (GPx). Finally, the mRNA expression of collagen type III (col3a1) and MMP13 was evaluated. Real-time qPCR was performed with an Abi Prism 7900HT Sequence Detection System (Applied Biosystems) using SYBR Green (Applied Biosystems). All samples were normalized to glyceraldehyde-3-phosphate dehydrogenase (GAPDH) or hypoxanthine-guanine phosphoribosyltransferase (HPRT). The 2^-ΔΔCT^ method was used to analyse the results **[47]**. All RT-qPCR results are expressed as mean ± SEM, and compared to expression levels of non-exposed group, which is set at 1. Primers were designed using Primer-BLAST software (http://www.ncbi.nlm.nih.gov/tools/primer-blast/). Sequences of the forward and reverse primers used are listed in Table 1.

**Table 1:** Primers sequences used for RT-qPCR. Primers sequences were obtained using Primer-BLAST.

Gene	Genebank accession n°	Strand	Sequence
Col3a1	NM_009930.2	Sense	5’TCCCTGGAATCTGTGAATC3’
		Antisense	5’TGAGTCGAATTGGGGAGAAT3’
Gapdh	NM_001289726.1	Sense	5’CCCCAGCAAGGACACTGAGCAAG3’
		Antisense	5’TGGGGGTCTGGGATGGAAATGTGA3’
Gpx1	NM_008160.6	Sense	5’CTGTGAACTCTTGTCAATG3’
		Antisense	5’AACTGTGTCAGGTATCTCC3’
Hmox1	NM_010442.2	Sense	5’GGGACTACACCGAGATGAACG3’
		Antisense	5’TCCGCAGGAAGGTAAAGAGC3’
Hprt	NM_013556.2	Sense	5’TCAGTCAACGGGGGACATAAA3’
		Antisense	5’GGGGCTGTACTGTTAACCAG3’
Icam1	NM_010493.2	Sense	5’TTCTCATGCCGCACAGAACT3’
		Antisense	5’TCCTGGCCTCGGACACACATTA3’
Ifng	NM_008337.3	Sense	5’TCTGGGTTCTCCTCCTGCGGC3’
		Antisense	5’GGCGCTGGACCTGTGGGTTG3’
Mcp1	NM_011333.3	Sense	5’GCACCAGCACCAGCAACTCT3’
		Antisense	5’TGGATGCTCCAGCCGGCAACT3’
Mmp13	NM_008607.2	Sense	5’ACAGGCTCCGAGAAATGCAA3’
		Antisense	5’CCACATCAGGCACTCCACAT3’
Nfe2l2	NM_010902.3	Sense	5’CGAGATATACGCAGGAGAGGTAAGA3’
		Antisense	5’GCTCGACAATGTTCTCCAGCTT3’
Sele	NM_011345.2	Sense	5’ATGCAGCGCACAAGGGCAGT3’
		Antisense	5’CCCGTGGCACCACACGTCAG3’
Tnf	NM_013693.3	Sense	5’GACAAGGCTGCCCCGACTA3’
		Antisense	5’AGGGCTCTTGATGGCAGAGA3’
Vcam1	NM_011693.3	Sense	5’AAGCCGGTCACGGTCAAGT3’
		Antisense	5’GGTCACCCTTGAACAGAGATCAATC3’

### Aortic protein expression and activity

Total aorta proteins were extracted according the manufacturer’s instructions (Total protein extraction kit, Millipore), and protein quantity was measured by a classic Bradford assay. To assess the expression of MMPs implicated in atheromatous plaque destabilization (MMP2, MMP3 and MMP8) in aorta, we perform a multiplex analysis on protein extracts according the manufacturer’s instructions (MMMP1MAG-79K, Merck Millipore). GPx activity was measured in aortic protein extracts according the manufacturer’s instructions (Glutathione Peroxidase Assay Kit, Cayman Chemical). GPx activity in tissues from non-exposed mice was set at 100%.

### Histological and immunohistochemical analyses of aortic plaque phenotype

Cryosections of 7 μm thickness were cut from the origin of the aortic root throughout the aortic sinus, for histological and immunohistochemical analysis. All images were acquired using an Axiophot (Zeiss). Mean lesion area and composition was calculated using Histolab software (GT Vision LTD) as described previously **[48, 49]**. Lesion area of control, unexposed mice was set as 100% relative to the total aortic sinus area. Quantification of the positive stain area of plaque components was calculated as a proportion of total lesion area, with values in control, unexposed mice set as 100%.

#### Lipid and collagen staining

Five to seven sections per animal were stained for the oil red O (Sigma-Aldrich) to evaluate the lesion area, and 5–7 sections per animal were stained with Picrosirius red (Sigma-Aldrich) to evaluate plaque collagen content.

#### Smooth muscle alpha-actin and macrophage immunostaining

VSMC and macrophage content were determined by immunofluorescence using monoclonal anti-α-smooth muscle cell actin (clone 1A4, Life Science A5691) and anti-CD68 (Abcam 1252), respectively. The aortic sinus cryosections were incubated then with primary antibody (1:100), rinsed, and further incubated with fluorescently labeled secondary antibodies (1:500) (Invitrogen). At least five sections per animal were stained. For negative controls, sections were incubated with secondary antibody only. Nuclei were stained with DAPI.

#### Superoxide and MMP activity staining

The evaluation of reactive oxygen species (ROS) production in the entire plaque area of the aortic sinus was performed by Dihydroethidium staining (DHE, 2 μM; Molecular Probes), applied during 30 minutes at 37°C. For negative control, sections were incubated with PBS 1X only. To assess MMP activity, aortic sinus cryosections were incubated at 37°C for 24 hours with fluorogenic gelatine substrate (DQ gelatine, Molecular Probes, Life Technologies) in a dark humid chamber. Slides were rinsed in PBS and nuclei stained with DAPI. Negative control sections were incubated without DQ-gelatine. All slides were independently examined on a blinded basis for the level of ROS or MMP staining, using a 0- to 4- point intensity gradient.

### Statistical Analysis

Experiments were made with 10 animals per group per experimental condition. To verify the normality, we performed a Shapiro-Wilk test. All data followed a Gaussian distribution. One-way ANOVA and *Student's t-test* was used to compare exposed and non-exposed animals results. All results are expressed as means ± SEM. Statistical software Sigma Plot 11.0 (SPSS) was used for all statistical analysis. Results with *p* < 0.05 were considered statistically significant.

## Results

### Chronic exposure to 137Cs has no effect on animal general health parameters or plasma lipid levels

**Fig 1:**
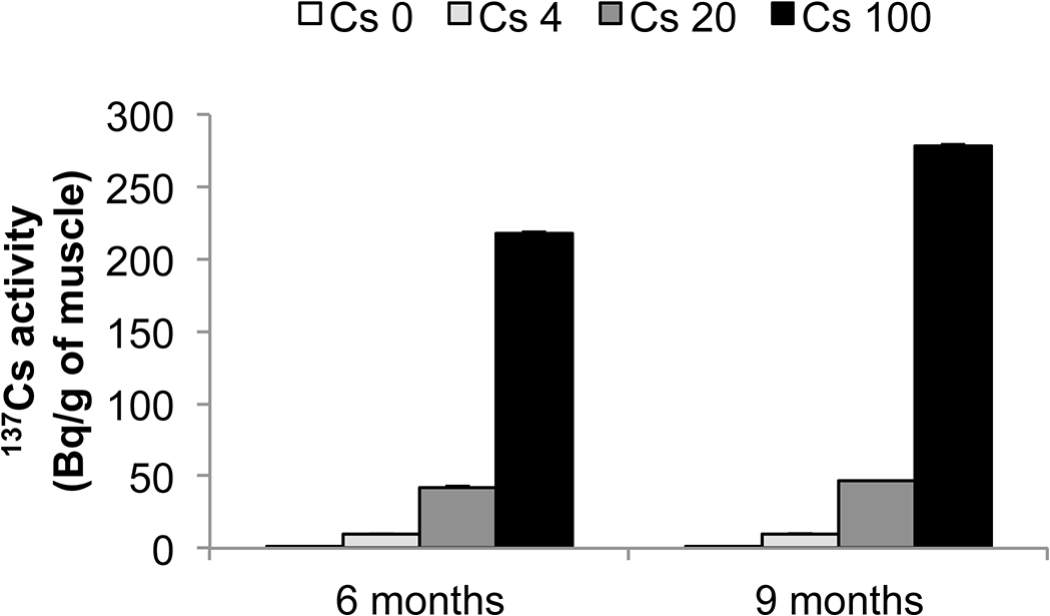
^137^Cs activity measurement correlates with ^137^Cs absorption in the gastrocnemus muscle, validating our experimental model. Activity is expressed as mean ± SEM of n = 8 per group per time.

**Fig 2:**
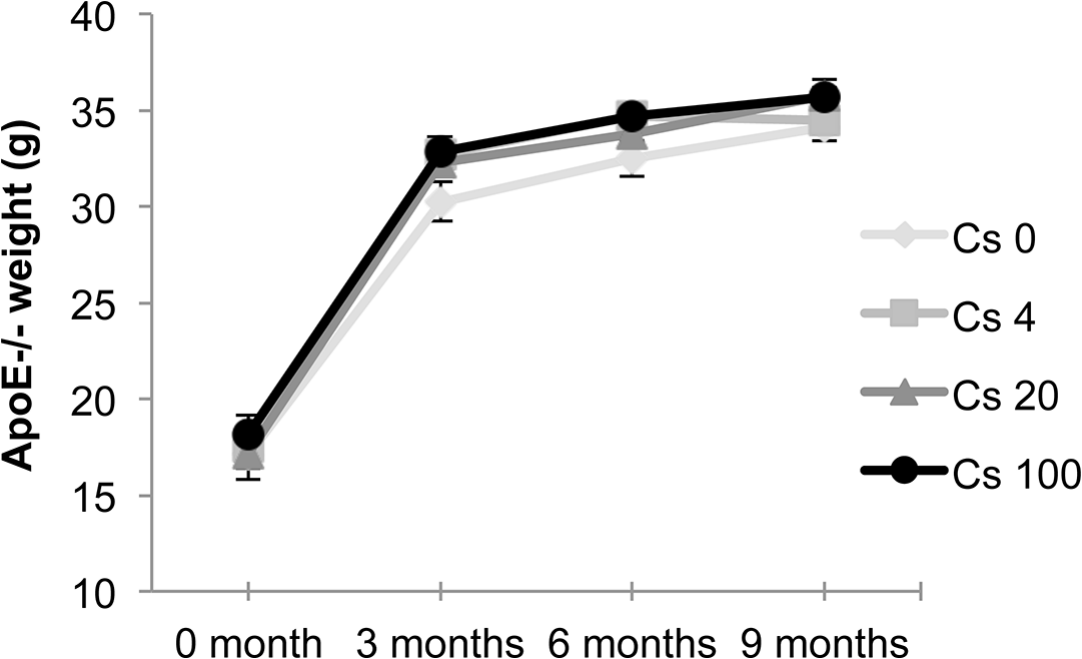
^137^Cs exposure does not interfere with weight gain in ApoE^-/-^ mice. Weight (in g) is expressed as mean ± SEM of n = 8 animals per group per time.

We found that ^137^Cs activity increased proportionally to ^137^Cs intake in the skeletal muscle of animals, where it accumulates preferentially (Fig 1 *)*. The activity expressed in Bq/g of tissue in the skeletal muscle after 6 months exposure was 0.12 ± 0.09; 8.00 ± 0.11; 37.58 ± 0.20; 191.91 ± 0.50 for 0, 4, 20 and 100 kBq/l respectively, and after 9 months exposure was 0.08 ± 0.08; 10.02 ± 0.15; 46.49 ± 0.49; 278.41 ± 1.00 for 0, 4, 20 and 100 kBq/l respectively. The resulting absorbed radiation doses due to ^137^Cs ingestion were calculated as previously described **[46]** and were 3, 15, and 75 mGy after 6 months and 6, 30, and 150 mGy after 9 months exposure to 4, 20 and 100 kBq/l of ^137^Cs, respectively. Exposure to ^137^Cs did not alter body weight in ApoE^-/-^ mice at any time over the 9 months of treatment, compared with unexposed mice (Fig 2). Likewise, plasma concentration of total cholesterol, HDL and LDL was equivalent in all groups irrespective of ^137^Cs concentrations (Table 2).

**Table 2:** Evaluation of cholesterol, LDL and HDL plasma content following 6 or 9 month exposure to ^137^Cs.

		Treatment			
		Cs 0	Cs 4	Cs 20	Cs 100
6 month exposure	Cholesterol (mmol/l)	11.89 ± 1.12	9.97 ± 1.91	9.08 ± 2.05	11.55 ± 1.80
	HDL (mmol/l)	3.38 ± 0.14	2.54 ± 0.39	2.92 ± 0.46	3.01 ± 0.38
	LDL (mmol/l)	8.10 ± 0.71	6.81 ± 1.64	6.31 ± 1.53	7.36 ± 1.43
9 month exposure	Cholesterol (mmol/l)	10.99 ± 1.62	9.30 ± 1.89	12.08 ± 0.67	13.88 ± 1.27
	HDL (mmol/l)	2.80 ± 0.29	2.47 ± 0.40	2.97 ± 0.10	3.21 ± 0.12
	LDL (mmol/l)	7.27 ± 1.15	6.36 ± 1.20	8.20 ± 0.36	9.32 ± 0.61

Chronic ^137^Cs exposure does not influence plasma lipid parameters. Results are expressed as mean ± SEM of n = 8 animals per group per time.

### Chronic exposure to 100 kBq/l 137Cs does not influence plaque size but it alters plaque inflammatory profile

**Fig 3:**
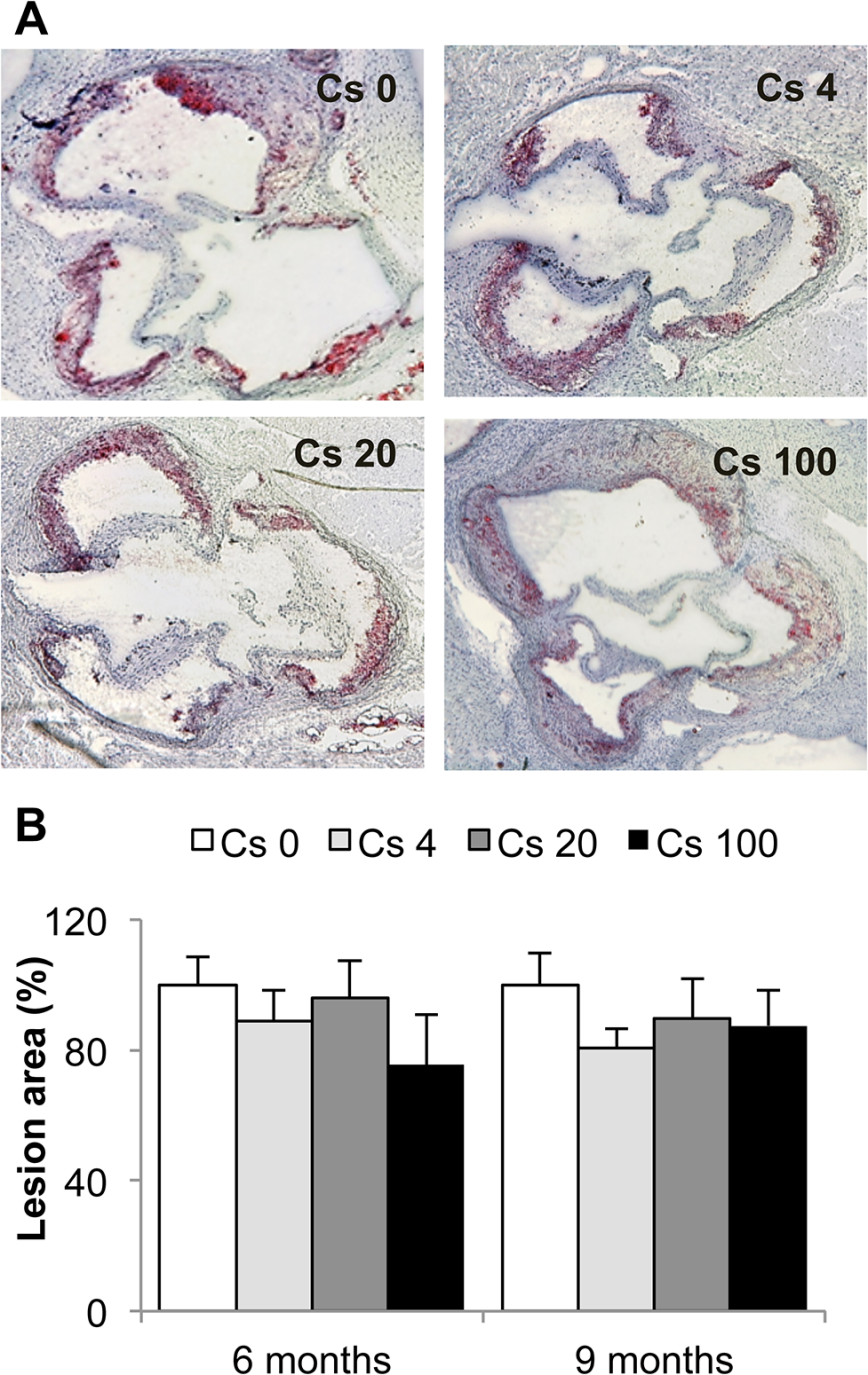
Atheromatous lesion area is not modified by a 6 or 9 month chronic internal exposure to ^137^Cs. **A:** Representative pictures of oil red O staining on aortic sinus cryosections after 6 months of exposure (magnification x 50). **B:** Quantification of the average lesion area performed using Histolab software. Lesion areas are expressed as mean ± SEM of n = 5 to 7 sections per animal.

**Fig 4:**
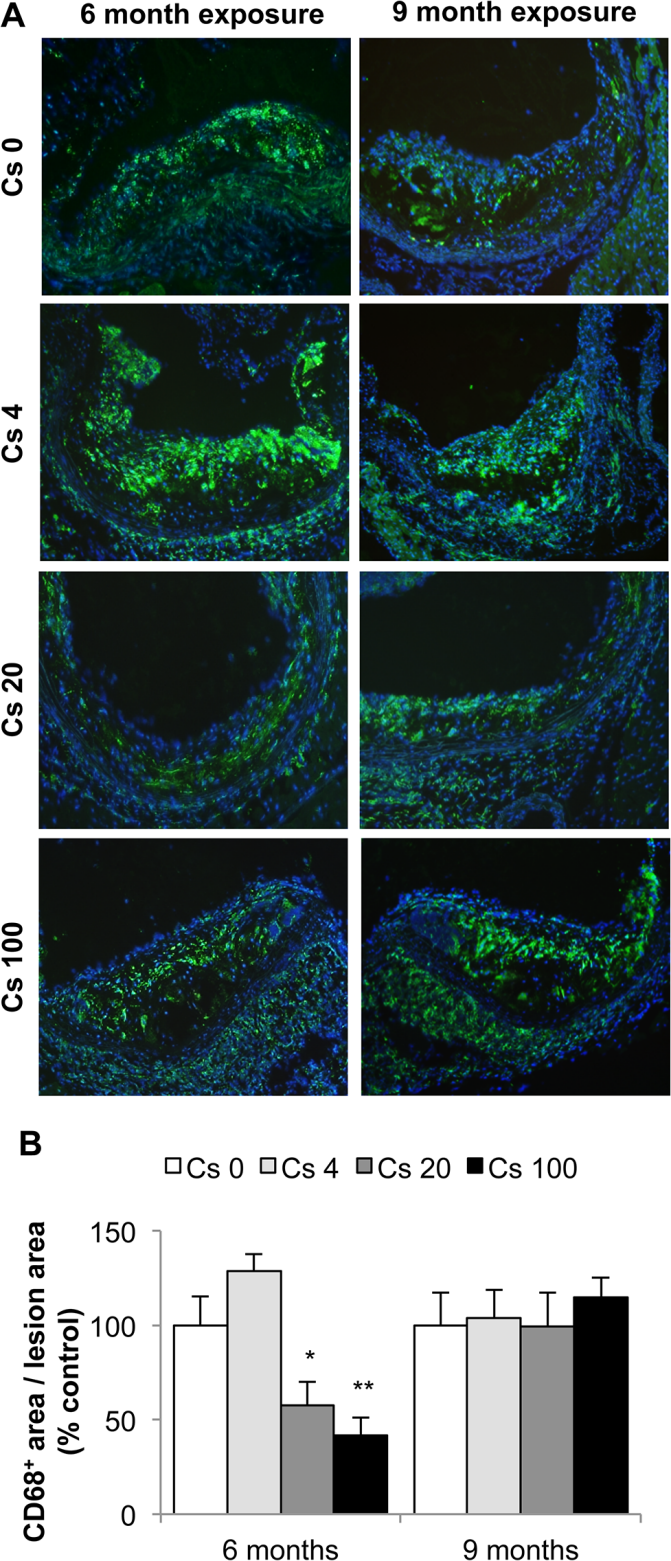
A six months exposure to 20 and 100 kBq/l ^137^Cs reduces macrophage content in atheromatous plaques. A: Macrophages were detected by CD68 immunostaining (CD68^**+**^ cells: green; nuclei: blue). Representative pictures obtained at magnification x100. B: Quantification of CD68^**+**^ cell content. Results are expressed as mean ± SEM of CD68^**+**^ surface area per plaque surface area, proportional to Cs 0 control group (%). *p<0.05, **p<0.01 vs non-exposed group. n = 5 sections per animal.

Atherosclerotic lesion size was measured in the aortic sinus of ApoE^-/-^ mice. No differences in plaque area were observed between ^137^Cs-exposed animals and non-exposed animals (Fig 3). Nevertheless, we investigated lesion composition in all groups. Macrophages are one of the major inflammatory cell types implicated in atherosclerosis progression and often the most abundant cells within lesions. To quantify macrophages within plaques, we performed a CD68 immunostaining. Our results show a decrease in CD68^+^ staining in lesions of mice exposed to 20 and 100 kBq/l ^137^Cs during 6 months (Cs 20: 54.0% ± 10.0%; Cs 100: 45.5% ± 7.7%) compared to non-exposed mice (Fig 4A and 4B). At the 9 month time point, these differences were no longer apparent (Fig 4).

**Fig 5:**
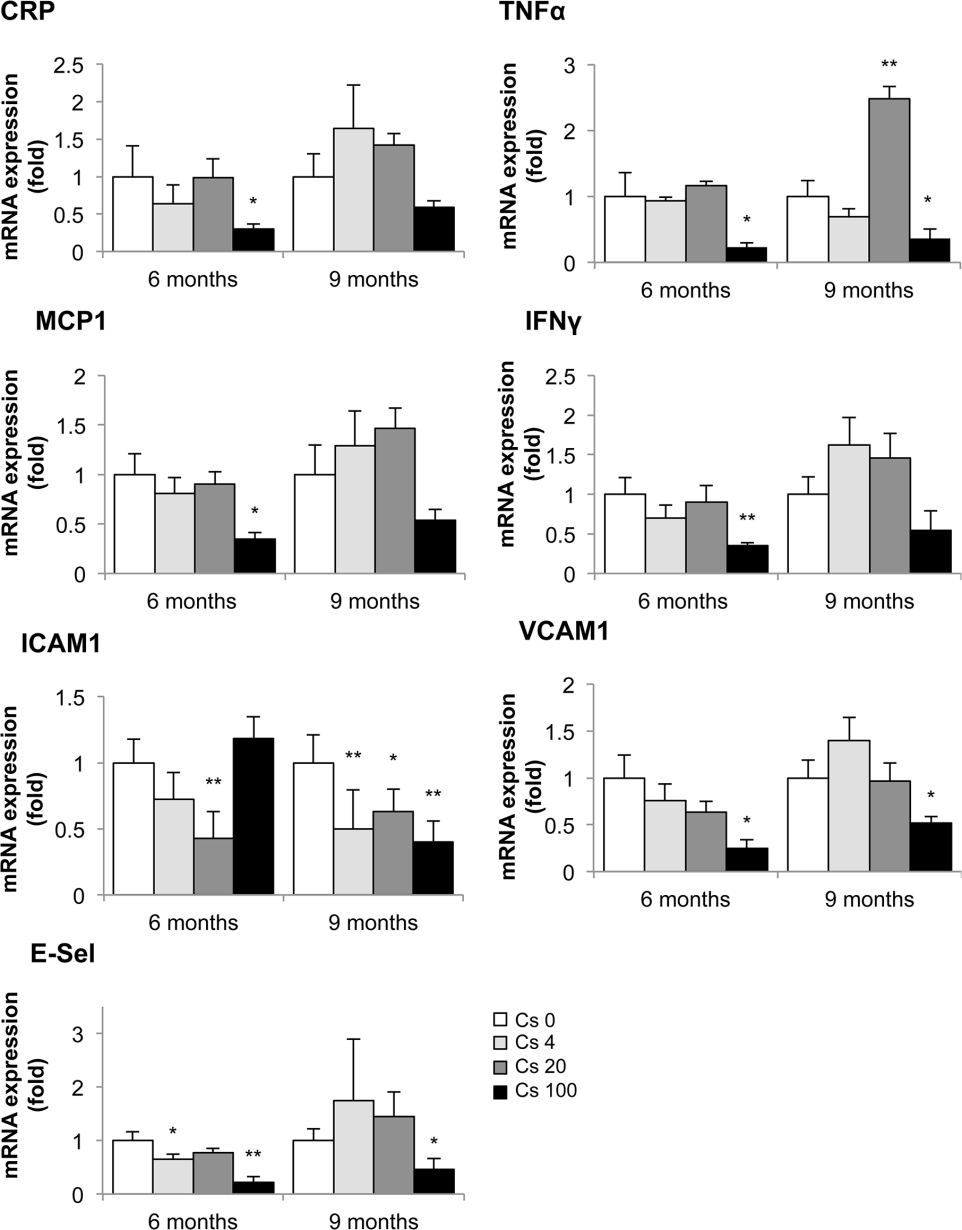
Aortic mRNA expression of inflammatory cytokines and adhesion molecules following a 6 or 9 month exposure to ^137^Cs. Levels of CRP, TNFα, MCP-1, IFNγ, ICAM-1, VCAM-1 and E-Sel were determined by RT-qPCR. GAPDH or HPRT were amplified and used as endogenous control. At 6 months, IFNγ and MCP-1 expression was significantly decreased in animals exposed to 100 kBq/l ^**137**^Cs compared to non-exposed animals; at 9 months, TNFα and ICAM-1 expression was significantly reduced. Results are expressed as mean of fold change ± SEM of n = 5–8 per group per time. *p < 0.05 and **p<0.01 versus Cs 0 control group.

Chemokine and adhesion molecule expression are key elements in the recruitment and diapedesis of monocytes in the vascular wall. Once inside the atherosclerotic lesion, most of these cells are transformed into macrophages. In keeping with the reduced macrophage content observed in plaques of mice exposed to ^137^Cs, we found a parallel decrease in the mRNA expression of many pro-inflammatory cytokines and adhesion molecules, measured by RT-qPCR, in their aortas (Fig 5). Specifically, aortic mRNA expression of CRP, TNFα, MCP-1, IFNγ, VCAM-1, and E-Sel were significantly decreased in ApoE^-/-^ mice exposed during 6 months to 100 kBq/l ^137^Cs (0.30 ± 0.07-fold, 0.23 ± 0.07-fold, 0.43 ± 0.13-fold, 0.21 ± 0.04-fold, 0.25 ± 0.09-fold, and 0.22 ± 0.10-fold, respectively, compared to un-exposed controls). TNFα, ICAM-1, VCAM-1, and E-SEL mRNA expression were also significantly decreased after 9 months ^137^Cs exposure (0.36 ± 0.14-fold, 0.40 ± 0.06, 0.52 ± 0.07-fold, and 0.46 ± 0.19-fold respectively). Although aortic MCP-1 mRNA was no longer lowered at 9 months, serum levels of the chemokine were significantly decreased after 9 months 100 kBq/l ^137^Cs exposure in ApoE^-/-^ mice compared to non-exposed animals (0.13 ± 0.06-fold, P<0.01). Hence, ^137^Cs exposure diminishes chemokine and adhesion molecule expression in ApoE^-/-^ mice, associated with reduced inflammatory cell content in atherosclerotic lesions.

### Exposure to 100 kBq/l 137Cs increases plaque stability in ApoE-/- mice

**Fig 6:**
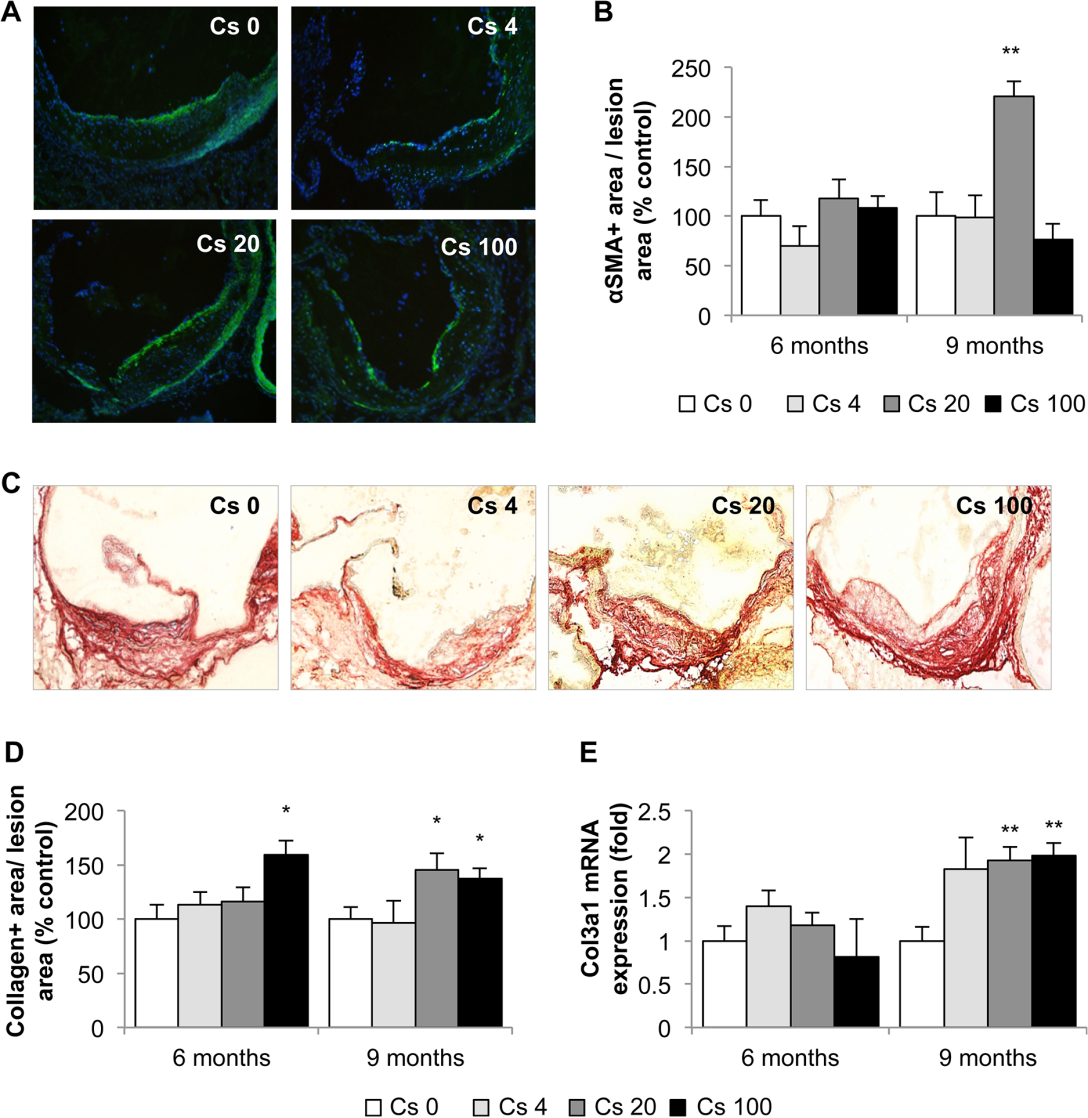
Indices of atheromatous plaque stability are enhanced after 9 months exposure to 100 kBq/l ^137^Cs. A: αSMA immunostaining for smooth muscle cells (αSMA^**+**^ cells: green; nuclei: blue, n = 5 sections per animal) and B: αSMA quantification within the plaques. No difference is observed after 6 months exposure, however, after 9 months exposure, a significant increase in αSMA^**+**^ area is noted for the group exposed to 20 kBq/l. C: Picrosirius red staining for collagen was performed on aortic sinus cryosections. Representative images obtained at magnification x100. n = 5 sections per animal. D: Quantification of collagen in the plaque. After 6 and 9 months of exposure to 100 kBq/l ^**137**^Cs, collagen content was increased in lesions. E: This result was paralleled by an increase in col3 mRNA expression, assessed by RT-qPCR, in the whole aorta. Results are expressed as mean ± SEM of n = 5–8. *p<0.05 vs Cs 0 control group.

The absence of effects of ^137^Cs on plaque size, despite diminished macrophage content, suggested that other lesion components might be affected by the treatment. Immunohistochemistry for α smooth muscle actin (αSMA), detecting smooth muscle cells (Fig 6A and 6B), and picrosirius red staining for collagen (Fig 6C) showed accentuated levels of both proteins in plaques from ApoE^-/-^ mice exposed to ^137^Cs. In fact, the 9 month exposure to 20 kBq/l doubled αSMA levels to 220.7 ± 15.4%, compared with non-exposed controls (100% ± 24.1; p<0.05). Similarly, quantification of picrosirius red revealed a significant increase in collagen within the plaques of mice exposed to 100 kBq/l ^137^Cs during 6 (159 ± 20%) and 9 months (137 ± 13%) compared with non–exposed animals (100 ± 14% and 100 ± 11%, respectively, at 6 and 9 months) (Fig 6C and 6D). Finally, type III collagen mRNA expression was also found to be enhanced 2-fold in the aorta of animals exposed to 20 and 100 kBq/l ^137^Cs during 9 months (p<0.05) compared to non-exposed animals (Fig 6*E*).

**Fig 7:**
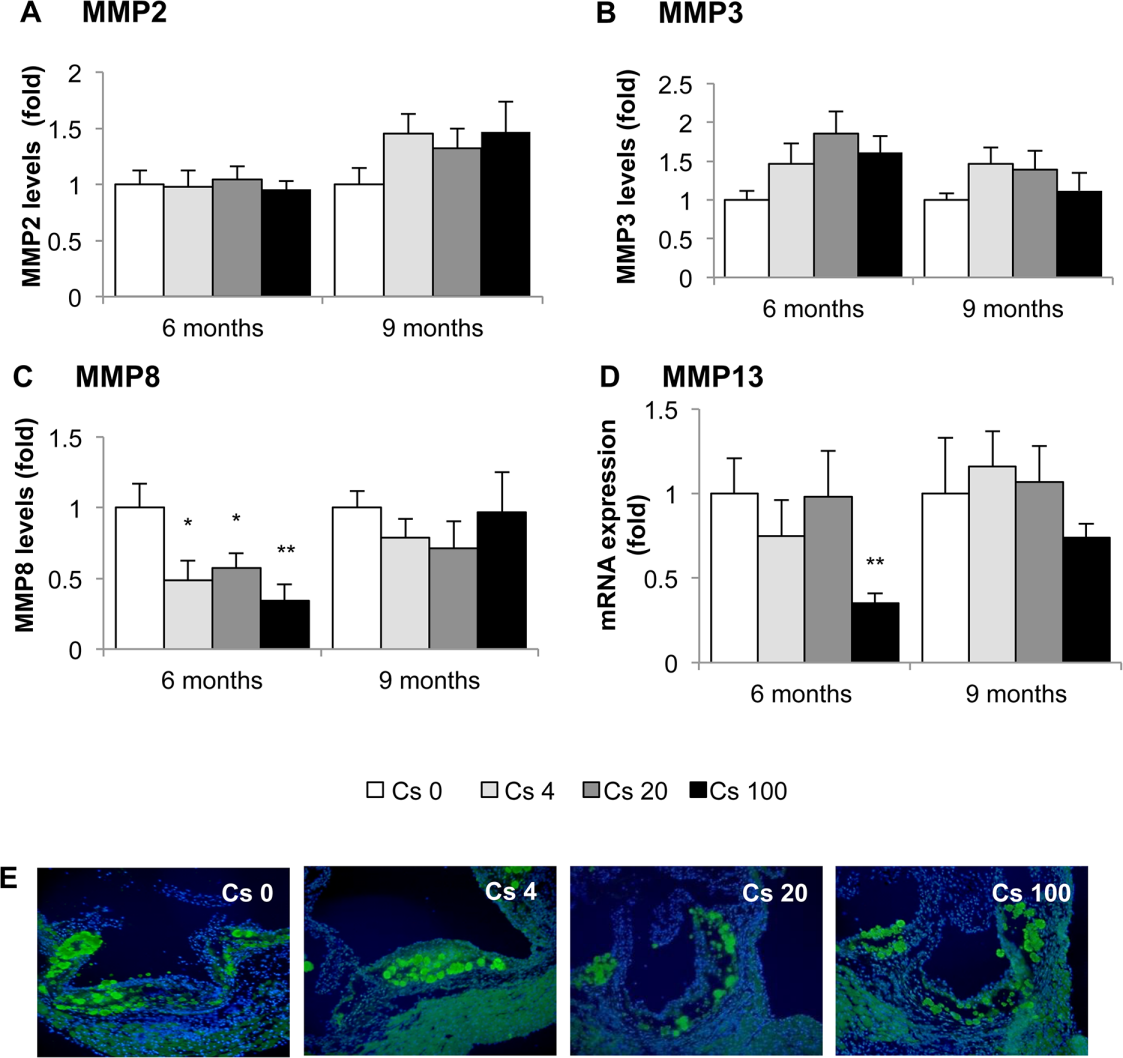
Aortic expression of MMP-2, -3, -8 and -13 mRNA after 6 or 9 months exposure to ^137^Cs. MMP aorta expression was evaluated using milliplex kit (for MMP2, -3 and -8) and by RT-qPCR for MMP13. A and B: No differences were observed for MMP2 and MMP3 expression levels at every ^**137**^Cs concentration and time exposure. C and D: concerning MMP8 and MMP13, we noticed a significant decrease for ApoE^**-/-**^ Cs100 after 6 months exposure. However, after 9 months, levels of MMP8 and -13 for this group are similar to control level. Results are expressed as mean ± SEM of n = 5–8 animals per group per time. **p<0.01 vs control. E: *In-situ* gelatinase activity, detected by enhanced fluorescence of fluorogenic gelatine substrate within the plaques and no difference were observed in MMP activity whatever the group.

In plaques, collagen is generally synthesized by smooth muscle cells and degraded by of matrix metalloproteinases (MMP) **[50]** Hence, we measured MMP2, MMP3 and MMP8 levels in aortic protein extracts by Milliplex assay, and determined aortic MMP13 mRNA expression by RT-qPCR. No differences in MMP2 or MMP3 expression were observed between the different groups (Fig 7A and 7B). However, we observed a significant decrease in both MMP8 (0.34 ± 0.04-fold) and MMP13 (0.33 ± 0.05-fold) in animals exposed to 100 kBq/l ^137^Cs during 6 months, compared with non-exposed animal (Fig 7C and 7D). Finally, we evaluated MMP2/MMP9 activity by in-situ gelatinase activity, and found no differences between groups (Fig 7E). In summary, ^137^Cs exposure enhances two parameters associated with increased plaque stability, collagen content and smooth muscle cell content.

### Exposure to 100 kBq/l 137Cs has little effect on redox balance in ApoE-/- mice

**Fig 8:**
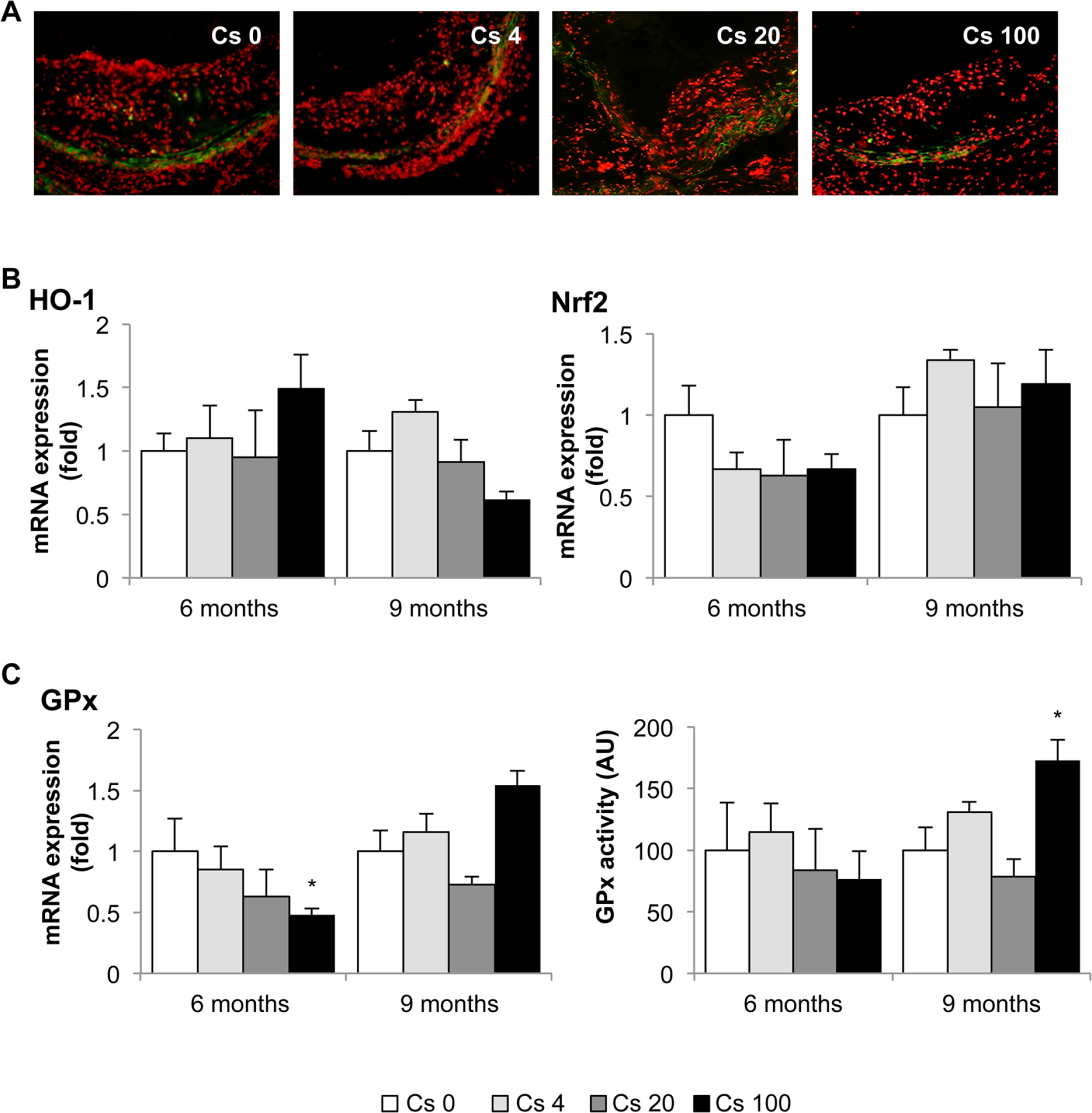
Changes in oxidative stress and related enzyme expression after 6 or 9 months exposure to ^137^Cs. A: Representative images of superoxide production within the atheromatous plaques evaluated by DHE staining after 9 months exposition (magnification x 200). n = 5 sections per animal. B: Qualitative analyses did not revealed any differences in aortic mRNA expression of HO-1 or Nrf2 between exposed or non-exposed groups, measured by RT-qPCR. C: However, aortic mRNA expression of GPx was reduced at 6 months in animals exposed to 100 kBq/l ^**137**^Cs. Moreover, GPx activity was significantly increased at 9 months, compared with non-exposed animals. Results are expressed as mean ± SEM of n = 5–8 animals per group per time. *p<0.05 vs Cs 0 control group.

Oxidative stress is a critical feature of atherosclerosis, contributing to endothelial dysfunction, LDL oxidation, upregulation of inflammatory pathways, and MMP induction. We first evaluated plaque superoxide production by dihydroethidium (DHE) staining. No differences were noted between the different groups of animals at that specific time points (Fig 8A). The aortic mRNA expression of HO-1 and Nrf2, two factors implicated in the oxidative stress response, was also equivalent in all mice (Fig 8B). However, mRNA expression of GPx, an anti-oxidative enzyme, was reduced after 6 months exposure to ^137^Cs at 100 kBq/l compared with non-exposed animals (0.48 ± 0.05-fold), but no significant difference was observed in GPx activity at this time point (Fig 8C). On the contrary, after 9 months exposure, GPx activity was increased in 100 kBq/l ^137^Cs-exposed animals compared with non-exposed animals (173 ± 32%), but GPx mRNA expression did not differ from controls (Fig 8C).

## Discussion

Our results show that chronic, low dose ingestion of ^137^Cs during 6 and 9 months had no consequences on plaque size and plasma lipid parameters. However, we observed a decrease in systemic pro-inflammatory cytokines expression, reduced plaque macrophage content, increased smooth muscle cells and collagen content, and lower MMP expression in ^137^Cs-exposed mice compared to non-exposed animals. Our data therefore indicate that exposure to ^137^Cs may actually promote a more stable plaque phenotype in mice.

Thus far, two studies tested the effects of chronic exposure to ^137^Cs, at doses comparable to post-accident exposure, on the cardiovascular system **[32, 42]**. These two studies yielded contradictory results. One reported alterations in the cardiovascular system of children who have lived in contaminated territories **[42]**, whereas the other showed subtle changes in blood pressure and in atrial expression of some genes, without any structural, pathological or clinical disorders, in an animal model **[32]**. Internal exposure to ^137^Cs during several months was previously reported to have no significant effects in different physiological systems (digestive system, central nervous system and immune system) in mice **[51–53]**. However, most experimental studies on irradiation and atherosclerosis exposed animals to external ionizing radiation for a short time. The results ranged from an increase in inflammation and atherosclerosis progression with high doses **[14],** to decreased atherosclerosis with very low doses administered at a low dose rate **[54]**. Interestingly, in most situations where inflammatory condition were modelled, the outcome of irradiation exposure was anti-inflammatory **[33–35, 55, 56]**. In vitro, low dose irradiation increased endothelial expression of ICAM-1 when delivered in fractionated episodes **[57]**, and low dose irradiation activated immune cells, orienting macrophages towards a pro-inflammatory phenotype **[58]**. However, low dose irradiation also decreased inflammatory cytokine production, reduced migration, and increased chemotaxis in macrophages, all of which could be linked to resolution of inflammation **[59]**. In summary, although some in vitro studies tend to link irradiation with pro-inflammatory processes, most animals studies describe a protective effect of low-dose radiation in the setting of inflammatory disease.

To the best of our knowledge, this is the first work investigating the effects of a chronic internal low dose exposure to ^137^Cs on atherosclerosis development and progression. In comparison, our study demonstrated that chronic, low-dose ^137^Cs induced no significant modifications in atherosclerotic plaque size. Our results are in line with those of *Mitchel et al* **[54].** They reported that a certain dose given at a low dose-rate (1 mGy/min) is protective against atherosclerotic lesion development whereas the same dose given at a high dose-rate (150 mGy/min) increases plaque size at late stages of the pathology. The importance of the dose-rate even after chronic low-dose exposure was confirmed in a model of premature senescence in cultured endothelial cells **[60].** Interestingly, the dose-rate during internal contamination, the chosen route of exposure in our study, is around 6 μGy/h, which is much lower than that used in previous works.

Our investigation of the effects of chronic ^137^Cs exposure revealed that indices of inflammation were actually reduced in exposed mice compared with non-exposed mice. Indeed, we observed a decrease in CRP, TNFα, MCP-1, and IFNγ, associated with a decrease in macrophage content within the plaques, after 6 months ^137^Cs exposure. Many of these effects persisted at 9 months. Our results are in accordance with some reports showing the anti-inflammatory effect of low-dose irradiation **[51, 61]** and of low dose radiotherapy (<1Gy or fractionated) **[62–64]**. Moreover, a previous study highlighted that the anti-inflammatory effect of low dose radiotherapy, which reduced adhesion of peripheral blood mononuclear cells to endothelium in vitro in the absence of effects on adhesion molecule expression **[65]**. However, chronic ingestion of 20 kBq/l had no impact on leukocyte or lymphocyte numbers **[38, 53]**. In our model, ^137^Cs exposure diminished expression of ICAM-1, VCAM-1 and E selectin, further contributing to the potential protective effects of exposure. Hence, the reduced levels of macrophages within the plaques of ^137^Cs-exposed mice was probably due to diminished pro-inflammatory cytokines (CRP, TNFα, MCP-1, IFNγ), but could also be partially explained by a reduction in adhesion molecules. After 9 months, TNFα, ICAM-1 and VCAM-1 expression was still attenuated in exposed mice, but macrophage content no longer differed between Cs-exposed and unexposed mice. This may be ascribed in part to local proliferation on CD68+ cells, as recently demonstrated **[66]**.

In our study, reduced plaque macrophage content after 6 months to ^137^Cs suggested a potential increase in stability. Interestingly, most clinical manifestations of atherosclerosis are related to plaque instability rather than lesion size. Vulnerable human atherosclerotic plaques are characterized by increased accumulation of macrophages, a large lipid pool, a thin fibrous cap, and decreased smooth muscle cell and collagen content; these plaques are more prone to rupture than stable plaques **[67–69].** Plaque rupture is an uncommon occurrence in murine models of atherosclerosis. However many morphologic features of atheroma that are prone to plaque rupture in humans can be seen in murine lesions **[70]**. Accumulated macrophages not only become foam cells that eventually become major constituents of the necrotic lipid core, they also synthesize and secrete matrix metalloproteinases which destroy collagen and thereby weaken the fibrous cap **[10, 71]**. Moreover, some reviews have emphasized the role of VSMCs in maintaining the integrity of plaque, in part through collagen synthesis, and suggested that VSMC proliferation may be beneficial to plaque stability **[72]**. Nevertheless, inflammation can inhibit collagen I and III production **[73]** and stimulate MMP production **[10]** by VSMC. In our study, we found that 6 months of ^137^Cs exposure was associated with reduced inflammatory mediators, lower CD68+ staining, and diminished expression of MMP8 and MMP13. At 9 months, plaque macrophage numbers were equivalent in exposed and non-exposed mice, but VSMC content was enhanced. In parallel, collagen content and collagen gene expression were increased. *Schiller et al* **[74]** demonstrated macrophage rich and collagen poor lesions in the aortic roots of irradiated LDLR^-/-^ mice after acute high dose (10 Gy) total body irradiation. Another group **[75]** also observed a decrease in VSMC and an increase in macrophages, coupled with higher levels of MMP8, in irradiated arteries. Thus, there truly appears to be a dose effect related to beneficial or adverse impacts of radiation on markers of plaque stability. Differences in levels of inflammation may very well underlie the opposite outcomes of high and low radiation exposure.

We hypothesized that changes in oxidative stress levels could account for the lower inflammatory profile of mice after ^137^Cs exposure. In a model of granulomatous disease, the anti-inflammatory effect of irradiation was correlated with an increase expression of oxidative stress parameters like HO-1 **[76]**. Similarly, it was shown that low doses of X-rays modulate the oxidative burst, which plays an anti-inflammatory role by reducing activated macrophages **[77]**. These observations could be attributed to hormesis, whereby a damaging agent causing a mild stress response results in a beneficial effect. Whereas high doses of radioactivity increase oxidative stress and inflammation significantly, low doses on the contrary modulate oxidative stress and inflammation **[66, 78, 79]**. However, in our experimental model we did not observe any striking difference in oxidative stress parameters after ^137^Cs exposure. The reduced expression of the anti-oxidant enzyme glutathione peroxidase (GPx) at 6 months was counterbalanced by increased GPx activity at 9 months. Moreover, plaque ROS status, measured by DHE staining, was not altered by ^137^Cs exposure. Hence, although our results do not discount a potential role for oxidative stress in the anti-atherogenic response to ^137^Cs at early time points, changes in oxidative stress are unlikely to explain the decreased in pro-inflammatory parameters and reduced macrophage content in plaques observed in mice exposed to ^137^Cs at 6 and 9 months.

The limitations of the current work include the use of young animals and reliance on the mouse model, in which plaques are not rupture-prone. Nevertheless, our study demonstrates that chronic low dose internal exposure to ^137^Cs, comparable to what is found in contaminated territories, does not potentiate atherosclerosis progression. On the contrary, we observed that such exposure enhances the stability of atherosclerotic plaques in ApoE^-/-^ mice, by inhibiting the expression of inflammatory cytokines and stimulating accumulation of collagen within the plaques. Further studies will be required to assess how early modification in the plaques of mice exposed to ^137^Cs may alter lesion composition at later time points, with special consideration for smooth muscle cell content (since VSMCs are the main cell type responsible for collagen synthesis in the plaque **[8]**) and inflammatory mediator expression (including TGFβ which is both anti-inflammatory and a stimulant of collagen production).
